# What are the factors influencing the aversion of students towards reptiles?

**DOI:** 10.1186/s13002-021-00462-z

**Published:** 2021-05-19

**Authors:** Moacyr Xavier Gomes da Silva, Franciany Braga-Pereira, Mikaela Clotilde da Silva, José Valberto de Oliveira, Sérgio de Faria Lopes, Rômulo Romeu Nóbrega Alves

**Affiliations:** 1 Programa de Pós-graduação em Etnobiologia e Conservação da Natureza (PPGEtno), Universidade Federal Rural de Pernambuco, Av. Dom Manoel de Medeiros, s/n – Dois irmãos, Recife, PE 52171-900 Brazil; 2 Departamento de Sistemática e Ecologia, Universidade Federal da Paraíba, João Pessoa, PB 58051-900 Brazil; 3 RedeFauna, Rede de Pesquisa Em Diversidade, Conservação e Uso da Fauna da Amazônia, Brazil, Philadelphia, USA; 4 Programa de Pós-graduação em Ecologia e Conservação, Universidade Estadual da Paraíba, Av. das Baraúnas, 351 – Bodocongó, Campina Grande, PB 58109-753 Brazil; 5 Departamento de Biologia, Universidade Estadual da Paraíba, Av. das Baraúnas, 351 – Bodocongó, Campina Grande, PB 58109-753 Brazil

**Keywords:** Ethnozoology, Human attitudes, Environmental education, Nature conservation

## Abstract

**Background:**

Reptiles form a paraphyletic group with significant roles for human society, including species that are considered important for food, medicinal and mystical use and as pets. Some species are considered to be aversive, whereas others are captivating among people. Aversion is an important factor which should be considered in the conservation policies of these animals. As such, here, we investigate the demographic, educational, perceptives and behavioural factors of students related to their aversion and non-conservationist attitudes directed towards different reptile species and evaluated the effect of educational exhibition of animals as a strategy of mitigating these attitudes.

**Methods:**

The data were obtained through forms on the aversion and conservation of reptiles represented by three species, a chelonian, a snake and a lizard. The form was given in two instances, before and after a visit to a private zoo (Museu Vivo Répteis da Caatinga), where the students had contact with the aforementioned species. A total of 133 students participated in the study, among these, 43 from elementary school (21 females and 22 males), 29 from high school (16 females and 13 males), 37 from university biology students (22 females and 15 males) and 24 university mathematics students (6 females and 18 males).

**Results:**

Among all evaluated species, snakes were considered to be the most aversive species. The aversion attitudes differed between the three evaluated species when correlated to age and type of university courses. However, this pattern did not differ between student sexes. Older students had a lower aversion to the chelonian compared to the younger ones, but for snakes and lizards, the aversion was high among students of all ages. The university biology students had a lower aversion compared to the university mathematics students for the three species. The recognition and handle of the tested species and previous visits to educational exhibitions of animals were negatively related to aversion. The comparative analysis of the forms applied before and after the visit to the Museu Vivo Répteis da Caatinga showed that this visit influenced the decrease of aversion, but not in non-conservationist attitudes, for which the attitude scores had always been low.

**Conclusions:**

We conclude that reptile aversion varies in accordance with the taxon, being snakes the most disliked by students. The visit to the educational exhibition of animals contributed to the reduction of the observed aversion. This is especially true when the acquisition of educational information about species is associated with practical activities which includes contact with the animals. Finally, the fact that non-conservationist attitudes had been low towards all species perhaps demonstrates a conservationist tendency even for the most aversive species.

**Supplementary Information:**

The online version contains supplementary material available at 10.1186/s13002-021-00462-z.

## Background

Humans have coexisted with other animals since their origin, establishing a series of interactions throughout their shared history [1]. From these interactions, ambivalent feelings emerged directed towards animals, which varies depending on the taxa involved and the type of relationship that humans have been maintaining with them [2]. Animals which attract the affinity of people are generally favoured by biodiversity conservation campaigns, for example, pandas and sea turtles [3]. On the other hand, species that provoke attitudes of aversion, or those that are considered being harmful, disgusting or dangerous, such as amphibians, spiders, scorpions, snakes and bats, are often pursued and killed when found [4–7]. These attitudes of aversion can be associated with the theory that humans have an evolved predisposition to associate certain animals with fear, such as spiders and snakes [8].

Notable examples of animals that elicit sympathy and strong aversion in humans can be found among the reptiles [7]. This group contains representatives that are considered being popular, such as the tortoise, often kept as pets in many areas [9], and unpopular and aversive animals such as snakes, which are often pursued and indiscriminately killed in many locations [5, 6]. The feeling of aversion can be triggered by many factors like disgust, fear and/or cultural aspects [10, 11]. Snakes illustrate this situation well, since many of them are venomous and snake bites may cause human deaths, consequently instilling fear in people [12]. Such aspects, as the detection mechanisms for these dangerous animals and the tendency to fear snakes, have evolved along with mammals, especially primates, including humans [8]. Furthermore, these animals are the targets of many negative symbolisms which contribute to the creation of negative conservation perspectives in some people [6, 7].

Detecting and avoiding venomous snakes may have contributed to the survival of early humans and primates, and some snakes have evolved a number of traits to either deter potential predators or to avoid being trampled, such as warning or aposematic signals like dorsal zigzag patterns, warm colours, triangular head shape and sounds [13–16]. So, humans may have inherited an evolved tendency to associate snakes or certain snake features such as the slithering motion with fear [8, 17, 18]. A greater frequency of phobias has been found specifically for the female sex, which may be explained by cultural or biological factors [19, 20]. Age can negatively influence the relationship between humans and fear, e.g. younger individuals generally have higher fear of animals. On the other hand, other fears decrease with age, such as fear of injections [21]. In addition, academic training has been found to significantly affect aversion towards commonly disliked species such as snakes, having people with training in biology presented a lower aversion when compared to those with no training in the area [22]. So, if the feelings related to animals are in part instinctive, another part is associated to the type of interaction between humans and reptiles which has been established over time, including ecological importance, functional uses and aesthetic and cultural aspects associated with fauna [23–25].

From an ethnobiological perspective, some studies have been contributing to the understanding of the factors influencing attitudes of aversion towards wildlife. Here, we promote an artificial encounter between human beings and some reptiles seeking to understand how people with different characteristics deal with aversion and how visiting educative exhibitions can influence aversive and non-conservationist attitudes. In detail, in this study, we analysed the relation of animal exhibitions and social factors on elementary, high school and university student attitudes directed towards reptiles.

Before the visit to the reptile museum, we applied a form to the students to verify (1) if aversion varies towards reptile taxa (chelonian, lizard and snake), (2) if the attitudes of aversion towards reptiles vary in students of different sex (3) and ages, (4) if university biology students have less aversion compared to university mathematics students, and (5) if student behaviours that promoted previous contacts with animals positively influence on student attitudes directed towards reptiles. Finally, we applied the same form to the same group of students before and after a visit to a reptile museum where live reptiles are exhibited to (6) evaluate the effect of visiting educative exhibitions of animals as a strategy of mitigating human aversion and non-conservationist.

## Methods

### Study area and target participants

For the data collection, two educational institutions were visited: the Escola Estadual de Ensino Fundamental e Médio Professor Itan Pereira and the Universidade Estadual da Paraíba, both located in the Campina Grande city, in the state of Paraíba, Brazil. Campina Grande has a territory of 593,026 km^2^ and 385,213 inhabitants (population density of 648.31 inhab/km^2^), of which 95,769 are students of basic education. The municipality has a human diversity index (HDI) of 0.720 [26].

This research was divided into two phases. The first phase of data collection for the research consisted of applying a form for elementary, high school and university students (*n*=133). Later, a second phase consisted of a visit to the Museu Vivo de Répteis da Caatinga, where after an educative class, the form was reapplied for the elementary school and high school students (*n*= 72).

Our complete sample consisted of 133 students: 43 from elementary school (21 females, 22 males), 29 from high school (16 females and 13 males), 37 university biology students (22 females and 15 males) and 24 university mathematics students (6 females and 18 males). The undergraduate courses do not provide specialisations in any field. The age of the interviewees varied from 12 to 29 years old. The students included in this study are not part of any specific ethnic group; all are inserted in the socio-cultural context of Brazilian urban communities, where the majority of the population is Christian, and have access to mass media (television, radio) and the Internet in general. The elementary and high school students have low income, and their families receive up to two minimum wages of R $2200, about US $ 379.10 per month.

The Museu Vivo de Répteis da Caatinga is located in the municipality of Puxinanã in the state of Paraíba, north-eastern Brazil (latitude. -7182014; longitude. -35.923545). The Museu Vivo de Répteis da Caatinga is a private zoo with both exotic and native reptile species to Brazil. The museum houses approximately 300 live animals, the majority of which are snakes. In this museum, lectures are offered to visitors about reptiles, with the aim of promoting ecological awareness and to discourage the indiscriminate killing of snakes [26].

This research was approved by the Ethics Committee of the Centro de Ensino Superior e Desenvolvimento-CESED/PB, with the certificate number CAAE 07989319.8.0000.5175 and licence number 3.201.771. Following the rules of this ethics committee, before applying the research form, a Terms of Free and Clear Consent (TFCC) was given to the students over the age of 18 and to the parents of students under the age of 18. In addition, a Terms of Assent (with a language easier to understand) was given for the students under the age of 18.

### Data collection

**Fig. 1:**
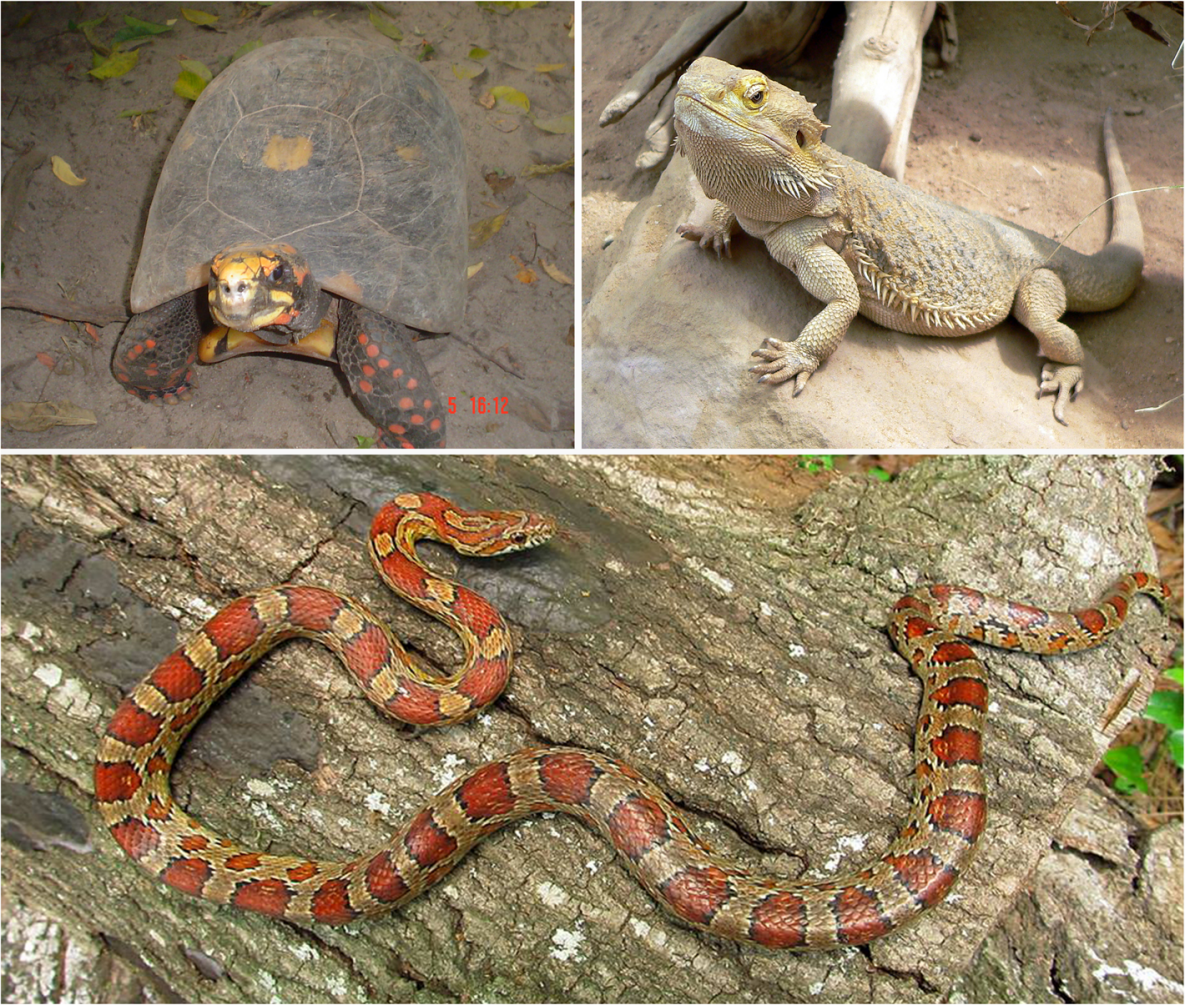
Species of reptiles used in the study. Above(left): Chelonian (*Chelonoidis carbonaria*) (Photo-Rômulo Alves); above(right): Lizard (*Pogona vitticeps*) (Photo - Frank C. Müller, via Wikimedia); below: Snake (*Pantherophis guttatus*) (Photo - Glenn Bartolotti, via Wikimedia)

Data collection was performed from January to June of 2019 through the completion of a form about three species of reptiles kept in the Museu de Répteis da Caatinga. The form was given twice for the students, once in their education institutes and other after visiting the museum. The animals selected were a chelonian—a tortoise, *Chelonoidis carbonaria* (Spix, 1824); a lizard—central bearded dragon, *Pogona vitticeps* (Ahl, 1926); and a snake—corn snake, *Pantherophis guttatus* (Linnaeus, 1766) (Fig. 1). We chose reptile species with the potential to elicit different levels of aversion, all presenting biological and behavioural characteristics that allowed for the handling by the students.

Part of the form consisted of questions on (i) the recognition of the tested reptile species, (ii) if the species is feared, (iii) whether the species is considered to be important, (iv) whether the species had been previously handled, (v) if the student has pets and (vi) and if the student has had previously visited an educational exhibition of animals. The other part of the form consisted of 20 sentences (10 of aversive and 10 of non-conservationist attitudes) for which the student could choose (on a scale ranging from 1 to 5) how strongly they agreed or disagreed with the sentence. For this, we present a Likert scale of 5 points [27] based on the level of agreement (completely agree = 5, agree = 4, unsure = 3, disagree = 2 and completely disagree=1). After the form was completed, we summated the levels of agreement of the ten sentences referring to attitudes of aversion and, separately, of the other five sentences referring to non-conservationist attitudes. Thus, for each interviewee, obtaining two scores which varied from 10 to 50 points (see an example in the Supplementary material, Fig. 1), which were used in our analysis.

The form was given pre-visit to all of the interviewees included in the study (elementary school, high school and university students). Subsequently, 72 elementary and high school students visited the Living Museum of Reptiles of the Caatinga, where they participated in a lecture on the snakes of the Caatinga and the practical exhibition of reptiles that involved the manipulation of some animals. During the lecture, morphological characteristics were presented to describe each species, and regional stories and ecological aspects were discussed aimed to reduce/combat snake killings. Two weeks after the visit, the same form (post-visit) was applied to the students who visited the museum, in order to assess the possible differences in terms of aversion and conservationist attitudes.

### Data analysis

We used cumulative link mixed models (CLMM) to compare the aversion and the non-conservationist attitudes of students directed towards different species. We chose CLMM because the data of aversion and the non-conservationist attitudes are ordinal, ranging from 10 to 50. For this model, we considered the student as a random variable whereas the species were considered as a variable of fixed effect. We also used CLMM to verify the relation of aversion of each species with the following predictor dichotomous (yes or no) variables: (i) the recognition of the tested reptile species, (ii) having previously handled the species, (iii) having previously visited an educational exhibition of animals, (iv) having pets, (v) fear the species and (vi) consider the species to be important. Before the analysis, we tested the co-linearity (*p >* 0.05) among all the predictor variables of fixed effect, and we did not find co-linearity. For the CLMMs, we compared the values of AIC between the above-described models and null models (without variables of fixed effect), and we only considered the models with values of ΔAIC>6 [28, 29]. All the inferential analyses were performed on R version 3.5.3 (R Core Team 2019) based on the packet ordinal [27].

In addition, we elaborate three different linear models to verify the relation of aversion towards each species and the (1) sex, (2) age and (3) type of university course (biology/mathematics) of the student. We also used linear models to verify the effect of visiting educative exhibitions of animals as a strategy of mitigating aversion and non-conservationist attitudes by the students. Once in each one of these linear model, there is just one predict variable we used frequentist statistics to evaluate the effect of the variable, in the case with the estimate and *p* value. All the inferential analyses were performed on R version 3.5.3 (R Core Team 2019).

## Results

Student aversion was found to be greater towards snakes compared to the chelonid and the lizard, for both younger students compared to the older and for the mathematics students compared to the biology ones. Furthermore, aversion was reduced when students had contact with the animals, especially aversion to snakes.

### Aversion and non-conservationist attitudes directed towards different reptile species

Our models showed that there was a significant variation of aversion towards the different reptile species, having the snake the higher aversion score (median of 21 score points), followed by the lizard (median = 20) and the chelonid (median = 14). There was no significant variation in non-conservationist attitudes of the students towards the species (*p>*0.05 and ΔAIC>6) (Table 1).

**Table 1:** Details of the cumulative linked models elaborated to verify the relation of aversion and non-conservationist attitudes directed towards different reptile species

Response variable	Predictor variables	Estimate	Std. error	*z* value	Pr(>|*z*|)		AIC	AIC null model	ΔAIC
Aversion attitudes	Chelonid: lizard	1.510	0.2438	6.214	5.15E−10	***	2475.08	2539.29	64.21
	Chelonid: snake	1.870	0.2551	7.342	2.11E−13	***			
Non-conservationist attitudes	Chelonid: lizard	0.3424	0.2847	1.203	0.2291		1297.2	1297.05	−0.15
	Chelonid: snake	0.551	0.2886	1.909	0.0562				

### Effect of age, sex and university course of students on aversion towards reptiles

The age demonstrated an effect on the aversion of the chelonian, with older students presenting the lowest aversion to the species, when compared to the younger students (*p*<0.01). This variation was not observed when considering the lizard and snake (*p*>0.05). In terms of sex, there was no significant variation when considering aversion directed towards the species (*p*>0.05). In terms of the university course, biology students had the lowest aversion to all of the tested species (median of score points for the chelonian, lizard and snake were 11, 17 and 18, respectively) when compared to mathematics students, especially in terms of snakes (median = 14, 20 and 26, respectively) (*p*<0.05) (Table 2).

**Table 2:** Details of all linear models elaborated to verify the effect of age, sex and university course on aversion to each reptile species

Response variable	Predictor variable	Estimate	Std. error	*z* value	Pr(>|*z*|)	
Aversion to chelonian	Age	−0.3835	0.08916	−4.302	3.37E−05	***
Aversion to lizard	Age	−0.0455	0.18435	−0.247	0.805	
Aversion to snake	Age	0.2752	0.2057	1.338	0.183	
Aversion to chelonian	Males: females	0.8182	0.78	1.049	0.296	
Aversion to lizard	Males: females	1.978	1.491	1.327	0.187	
Aversion to snake	Males: females	1.892	1.687	1.122	0.264	
Aversion to chelonian	Biology: mathematics	19.899	0.7561	2.632	0.0108	*
Aversion to lizard	Biology: mathematics	5.135	2.076	2.473	0.0163	*
Aversion to snake	Biology: mathematics	9.595	2.231	4.301	6.73E−05	***

### Effect of student’s perceptions and behaviour on her/his aversion towards reptiles

We found that the aversion towards all the species considered was significantly related to student’s fear: the greater the fear, the greater the aversion (*p>*0.05). The attitude of aversion decreased significantly (*p*<0.05) (i) for the chelonid—in relation to the previously visited educational exhibition of animals; (ii) for the lizard—with the increase of recognition of the species and of perception of the species importance; and (iii) for the snake—for those students who have previously handled the animal. All students involved in this research recognise the chelonid, and so, this predictor variable was not considered in the model for this species (Table 3).

**Table 3:** Details of the cumulative linked models elaborated to verify the effect of student’s perceptions and behaviour on her/his aversion towards the reptiles

Response variable	Predictor variable	Estimate	Std. error	*z* value	Pr(>|*z*|)	
Aversion to the chelonian	Fear of the test species	3.085	1.022	3.018	0.00255	**
	Considers the test species to be important	−0.6114	0.57564	−1.062	0.2882	
	Handled the test species	0.09372	0.40163	0.233	0.8155	
	Has a pet animal	−0.0391	0.33802	−0.116	0.90783	
	Previously visited an animal educational exhibitions	−0.8038	0.31957	−2.515	0.0119	*
Aversion to the lizard	Recognise the test species	−0.7738	0.3843	−2.013	0.0441	. *
	Fear of the test species	28.585	0.427	6.694	2.17E−11	***
	Considers the test species to be important	−1.235	0.5255	−2.352	0.0187	*
	Handled the test species	−0.4026	0.5939	−0.678	0.4979	
	Has a pet animal	−0.351	0.3618	−0.97	0.3321	
	Previously visited an animal educational exhibitions	−0.6315	0.3391	−1.862	0.0625	
Aversion to the snake	Recognise the test species	−0.2571	0.8915	−0.288	0.7731	
	Fear of the species	33.089	0.437	7.572	3.66E−14	***
	Considers the test species to be important	−0.3144	0.7138	−0.44	0.6596	
	Handled the test species	−1.023	0.4186	−2.446	0.0144	*
	Has a pet animal	0.3848	0.3625	1.061	0.2886	
	Previously visited an animal educational exhibitions	−0.4529	0.3231	−1.402	0.1609	

### Effect of visiting the museum on aversion and non-conservationist inclinations

**Fig. 2:**
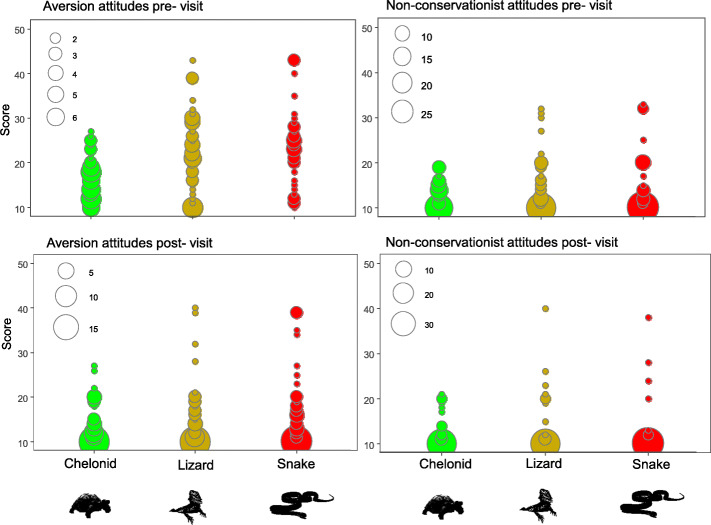
Aversion and non-conservationist attitudes pre- and post-visit to the Museu Vivo de Répteis da Caatinga towards the chelonian (*Chelonoidis carbonaria*), lizard (*Pogona vitticeps*) and snake (*Patherophis guttatus*) species. The sizes of the circles are proportionate to the number of students that gave each score for each species

The visit to the museum (that includes educative lectures and handling of live animals) significantly reduced attitudes of aversion on all reptile species. The decrease in aversion towards lizards stood out, with an initial median value of 22 reduced to 11. The visitation also decreased the non-conservationist inclinations, although this reduction was not significant (*p>* 0.05) (Fig. 2; Table 4). However, this is maybe because the scores regarding non-conservationists attitudes were low for all species in both pre- and post-visit (see details in Fig. 2).

**Table 4:** Details of all linear models elaborated to verify the effect of visiting the museum on aversion and non-conservationist inclinations. The estimated values indicate the coefficient associated with the listed variable on the left

Response variable	Predictor variable	Estimate	Std. error	*z* value	Pr(>|*z*|)	
Aversion to tortoise	Pre: post-visitation	−1.67	0.7722	−2.169	0.0315	*
Aversion to lizard	Pre: post-visitation	−5.74	1.42	−4.026	8.47E−05	***
Aversion to snake	Pre: post-visitation	−5.92	1.59	−3.704	0.000285	***
Non-conservationist attitudes—tortoise	Pre: post-visitation	−0.00505	0.486433	−0.01	0.992	
Non-conservationist attitudes—lizard	Pre: post-visitation	−0.2369	0.895	−0.265	0.792	
Non-conservationist attitude—snake	Pre: post-visitation	−0.8315	0.8686	−0.957	0.34	

## Discussion

The reptiles, while at the same time presenting ample useful ecological functions, can also be seen as harmful animals and can be associated with evil, in Brazil and in other parts of the world [30–33]. Nevertheless, this bad reputation varies depending on the taxon in consideration [7, 32], which was observed in our research. The greatest aversion was directed towards the snake when compared to the other studied reptiles (lizard and chelonian). A stronger aversion towards the snake was expected, since in many regions of the world, it is common for people to associate snakes to negative symbolisms, in addition to fear them (because of the risk that venomous snakes represent) and because of an evolutionary tendency of aversion in primates to aposematic signals that are commonly displayed by snakes [8, 13, 31]. On the other hand, chelonids such as tortoises, elicit more empathy in people, and they are popular pets in Brazil [30–32], which explains the low aversion observed towards this animal in our study and why all students recognised the species. In addition, these animals do not represent risks such as snakes, they are docile and have many useful purposes [31, 32], which positively influences the affinity of people towards them. Lizards, although they are not charismatic like tortoises, they are not disliked like snakes [32], a tendency which was also observed in our results. However, some lizards elicit aversion in Brazilians as they are considered to be dangerous, such as *Polychrus acutirostris* (Spix, 1825), although on a lesser scale when compared to snakes [32]. Furthermore, some lizards have similar morphologies to snakes, which contributes to people demonstrating aversion towards them [31, 34]. In another perspective, the triangular aposematic signs present on the body and shape of the serpent’s head would be the inducers of human beings’ aversion to snakes [13]. Thus, the animals’ triangular shapes, more present in the snake and the lizard, when compared to the tortoise, may have influenced our findings, justifying the greater aversion of the participants to the snake and the lizard. Kellert and Berry [35] emphasised that sex influences almost all the dimensions of attitudes and knowledge on animals and suggested that men and women have different emotional and cognitive orientations towards animals. This tendency is reinforced by diverse studies which point towards the influence of sex when considering aversion directed towards fauna [23, 36–38]. Our results, however, show that there was no difference in the level of aversion towards the three study species in terms of student sex. This is an indication that the culture of aversion to these animals is equally widespread between the two sexes, which was expected for snakes, which is generally disliked by the majority of the population in the study region and is feared and persecuted by the population in general. However, from these finds, we also highlight the importance to consider the influence of culture, and not only biological aspects, on aversion towards species.

Regarding the relation between the aversion and the students’ age, we found that older students showed the lowest aversion to chelonian; however, when related to the aversion towards lizards and snakes, we did not find any influence of the age. The result of older students showing lower aversion to animals was also observed in others studies. Campos et al. [39], for example, reported that increasing age increases familiarity with organisms and also influences attitudes towards them. This is a somewhat predictable situation, considering the development of rationality during the life of the human being, through all forms of cultural processes experienced [40, 41].

When considering the results obtained for the university students, it was evident that the academic training of students influences the aversion of the analysed species. As expected, a lower level of aversion was observed in the university biology students when compared to mathematic students. It is common that people who undertake courses such as biological sciences have a greater interest in themes associated with nature and are more favourable towards the conservation of fauna. Furthermore, during the course, students have access to information about fauna and its importance. This contributes to the reduction in student aversion, even when considering less popular animals and demystifies contexts which contribute to the aversion associated with these animals. Polák et al. [22] also affirmed the importance of training in biology as a factor which influences affinity towards snakes. Along the same lines, Oliveira et al. [37] suggest that attitudes of affinity with fauna are expected to be positively influenced by the increase in participation of formal and informal educational activities. This is due to the cumulative effect of the taught content which culminates in a greater understanding of fauna and contributes to the demystification of views which influence the aversion of people towards certain species.

The practice of visiting educational exhibitions of animal influenced the decrease in student aversion towards the tortoise and the lizard but not towards the snake. Visits to spaces such as museums and oceanariums can positively alter attitudes associated with fauna [42], as these spaces are areas of non-formal education where people construct and reconstruct their knowledge and perceptions of wildlife [43, 44]. They therefore represent areas that permit experience with nature and/or living creatures which can positively affect the affinity of humans towards animals [42–45]. In our research, we observed that the influence of the visit to the reptile museum on reducing aversion attitudes varied depending on the taxon, being less positive when considering less charismatic animals such as snakes, and revealing that aversion towards these animals must be combatted through varying educational strategies. The results obtained in our study indicate that handling is negatively related to snake aversion, that is, the manipulation of animals contributes to diminishing aversion. This observation is in accordance with the authors such as Ballouard [46] and Prokop [47], who indicated that the handling of unpopular animals decreases disgust and fear, considered as aspects which contribute to aversion. In addition, previous research reinforces that educational interventions, especially involving practical classes with lecture and manipulation of the animals, are important in improving knowledge and decreasing aversion towards snakes in students [32, 42–44].

Our results are also in accordance with some studies carried out in similar contexts, performed in different countries such as Japan [48], China [45] and Norway [49], where they demonstrated the positive effects of interactions with nature on human attitudes towards living creatures and natural ecosystems. Even more important, we presume that the influence of the visit promoted during this research will persist over time, may be reported to students’ relatives and friends and the aversion is unlikely to return to the initial level even in contact with aversive people. This is because some studies have been showing long-term effects of environmental education school activities [49] and that children learn and retain conservation principles in school environments and transfer them to their parents [49]. In addition, Vaughan [50] theorise that parents learned from children, and both groups transmitted course information to neighbours.

The conservationist inclinations of the students did not differ between the groups of reptiles, and the non-conservationist attitudes were low towards all species, which demonstrates a conservationist tendency even for the most aversive species. Because the scores regarding non-conservationists attitudes were low for all species in both pre- and post-visits, although the visitation to the museum also decreased the non-conservationist attitudes, this reduction was not significant. However small, this reduction is in line with previous studies which indicated that conservation efforts of non-charismatic animals, as is the case with some reptiles, are directly related to the scientific knowledge of people [50, 51]. Thus, educational strategies that increase scientific knowledge, especially those that involve direct contact with animals, appear to be a coherent alternative for increasing adhesion to the conservation policies of non-charismatic species, such as snakes, making these policies more efficient.

## Conclusion

Our results reveal that reptile aversion varies in accordance with the species in consideration, with snakes provoking the greatest aversion in students. We did not observe levels of aversion to vary with student sex, unlike what has been observed in studies which involve multiple taxa. We observed that older students had a lower aversion to the chelonian compared to the younger ones and that the university biology students had a lower aversion compared to the university mathematic students for all species included in this study. This may reflect the greater degree of knowledge of species and their importance held by biology students, demystifying, in some cases, negative views associated with fauna. Similarly, visits to reptile exhibitions and the handling of species by students contribute positively to the attenuation of aversive attitudes towards them. Finally, the fact that non-conservationist attitudes had been low towards all species perhaps demonstrates a conservationist tendency even for the most aversive species.

## Supplementary Information

**Additional file 1.** (Form provided to verify the influence of practical exposure on the attitudes directed towards reptiles).

## Data Availability

All data generated or analysed during this study are included in this published article.
