# Supplementary material for: What are the factors influencing the aversion of students towards reptiles?
Source: J Ethnobiol Ethnomed. 2021 May 19;17:35. doi: 10.1186/s13002-021-00462-z (PMC8136183; doi:10.1186/s13002-021-00462-z)
Supplement: Supplementary file 1 — Additional file 1. (Form provided to verify the influence of practical exposure on the attitudes directed towards reptiles). [file 13002_2021_462_MOESM1_ESM.docx]

FORM PROVIDED TO VERIFY THE FACTORS INFLUENCING THE AVERSION OF STUDENTS TOWARDS REPTILES

**SCORE 1: . SCORE 2: .**

**Educational Institute: .**

**Grade/Class: Level: ( ) Elementary School ( ) High School ( ) University**

**Volunteer:**

**Age: Sex: ( ) M ( ) F**

**Photograph Nº**

1. Do you know this animal? ( ) Yes ( ) No

If yes, what is the name of this animal_____________________________________?

1. Have you ever visited live educational exhibitions of animal, zoos, gardens, museums or other? ( ) Yes ( ) No

If yes, which _______________________________________ _?

1. Do you have a pet animal? ( ) Yes ( ) No

If yes, which _?

1. Have you ever handled this animal? ( ) Yes ( ) No

If yes, where ?

1. Are you scared of this animal? ( ) Yes ( ) No

Why_____________________________________________________________ ?

1. Do you consider this animal to be important? ( ) Yes ( ) No

Why_____________________________________________________________?

## Check where you agree with the statements.

## (information not in the form provided to the student: the follow 10 statements are related to aversive attitudes)

1. I would not touch this animal.


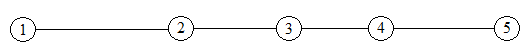


Completely agree Agree Unsure Disagree Completely disagree

1. I would not touch this animal, even if it was dead.


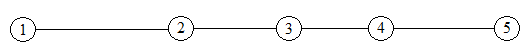


Completely agree Agree Unsure Disagree Completely disagree

1. If I saw this animal in a cage, I would be scared.


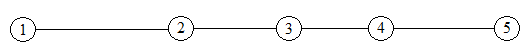


Completely agree Agree Unsure Disagree Completely disagree

1. If I saw this animal in person outside, I would be scared.


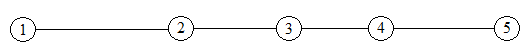


Completely agree Agree Unsure Disagree Completely disagree

1. Even thinking about this animal scares me.


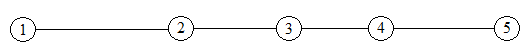


Completely agree Agree Unsure Disagree Completely disagree

1. This animal is ugly.


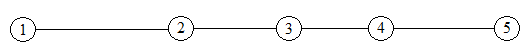


Completely agree Agree Unsure Disagree Completely disagree

1. This animal is disgusting.


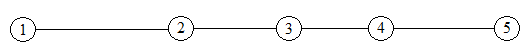


Completely agree Agree Unsure Disagree Completely disagree

1. I would not work with this animal.


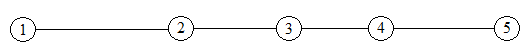


Completely agree Agree Unsure Disagree Completely disagree

1. If the teacher brought this animal alive to class, I would prefer to leave the class.


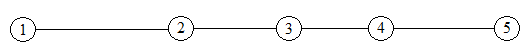


Completely agree Agree Unsure Disagree Completely disagree

1. This animal represents risks, danger.


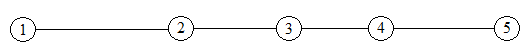


Completely agree Agree Unsure Disagree Completely disagree

## (information not in the form provided to the student: the follow 10 statements are related to non-conservationist attitudes)

1. It is not important to protect this animal.


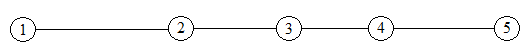


Completely agree Agree Unsure Disagree Completely disagree

1. This animal should not exist.


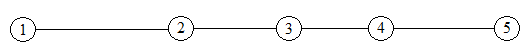


Completely agree Agree Unsure Disagree Completely disagree

1. This animal is useless


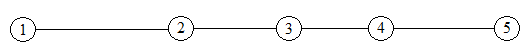


Completely agree Agree Unsure Disagree Completely disagree

1. This animal should be dead.


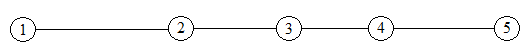


Completely agree Agree Unsure Disagree Completely disagree

1. This animal should be extinct.


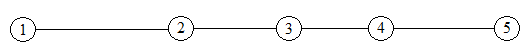


Completely agree Agree Unsure Disagree Completely disagree

1. This animal should not be protected.


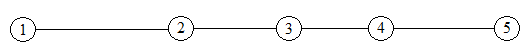


Completely agree Agree Unsure Disagree Completely disagree

1. The world would be better without this animal.


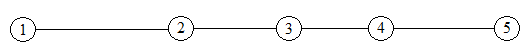


Completely agree Agree Unsure Disagree Completely disagree

1. I do not need this animal therefore, it should not exist.


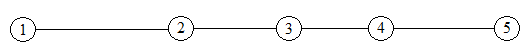


Completely agree Agree Unsure Disagree Completely disagree

1. Nothing or no one needs this animal, therefore it should not exist.


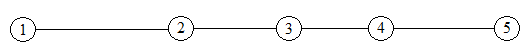


Completely agree Agree Unsure Disagree Completely disagree

1. If I could, I would eliminate this animal.


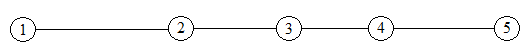


Completely agree Agree Unsure Disagree Completely disagree

#
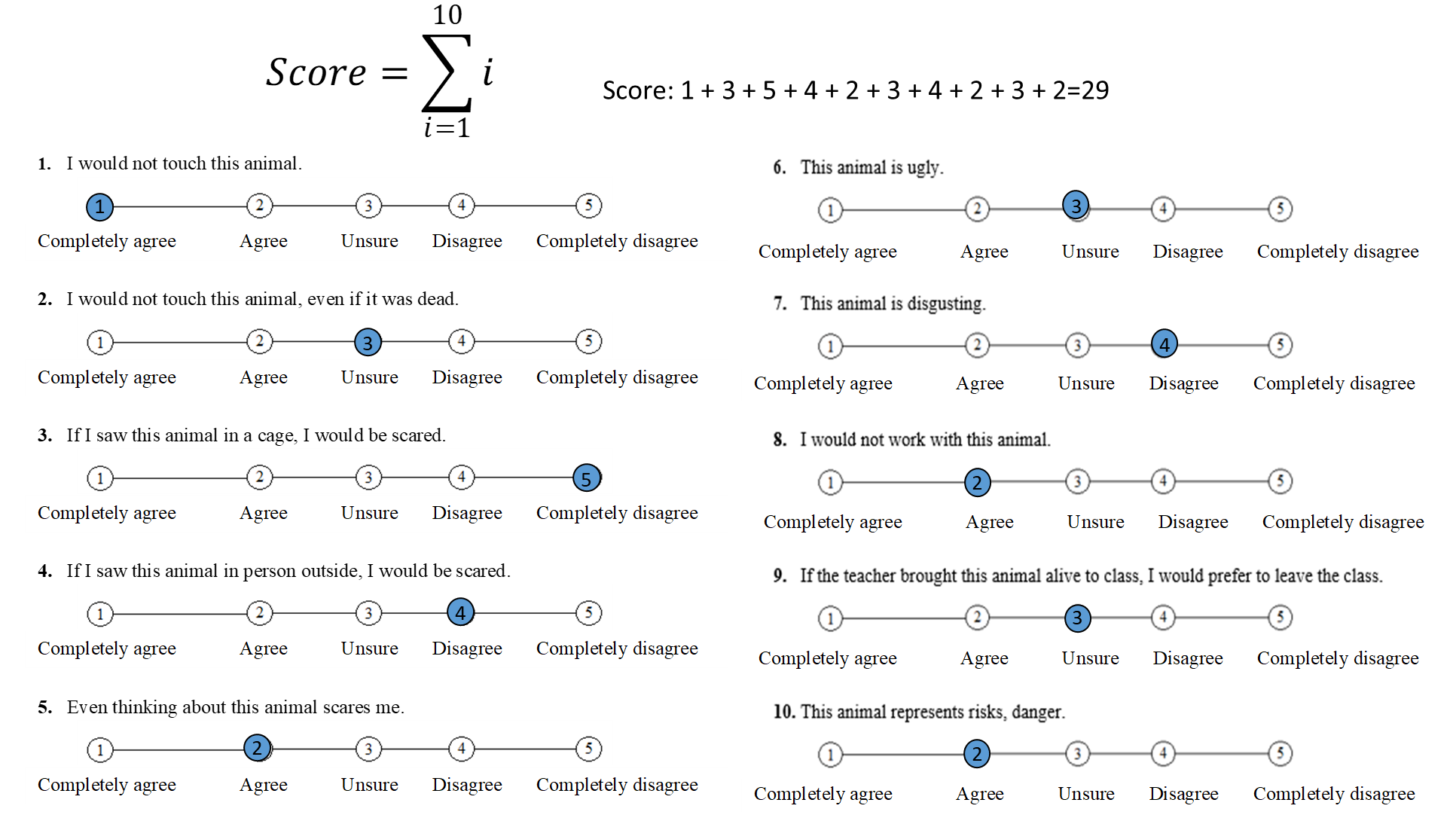


# **Figure 1.** example of how the final aversion score was calculated
